# Syntheses, crystal structures and Hirshfeld surface analysis of 2-(benzyl­sulfan­yl)-5-[4-(di­methyl­amino)­phen­yl]-1,3,4-oxa­diazole and 2-[(2-chloro-6-fluoro­benz­yl)sulfan­yl]-5-[4-(di­methyl­amino)­phen­yl]-1,3,4-oxa­diazole

**DOI:** 10.1107/S2056989023004164

**Published:** 2023-05-19

**Authors:** Rasul Ya. Okmanov, Abdukhakim A. Ziyaev, Azimboy Sh. Abdukarimov, Turdibek T. Toshmurodov, Tursunali S. Kholikov

**Affiliations:** a S. Yunusov Institute of the Chemistry of Plant Substances, Academy of Sciences of Uzbekistan, Mirzo Ulugbek Str. 77, Tashkent, 100170, Uzbekistan; b National University of Uzbekistan named after Mirzo Ulugbek, University Str. 4, Tashkent, 100174, Uzbekistan; Indian Institute of Science Education and Research Bhopal, India

**Keywords:** synthesis, 1,3,4-oxa­diazole, crystal structure, Hirshfeld surface analysis

## Abstract

The title compounds were synthesized by alkyl­ation of 5-[(4-di­methyl­amino)­phen­yl]-1,3,4-oxa­diazole-2-thiol with benzyl chloride or 2-chloro-6-fluoro­benzyl chloride in the presence of potassium carbonate. The yields of 2-(benzyl­sulfan­yl)-5-[4-(di­methyl­amino)­phen­yl]-1,3,4-oxa­diazole, C_17_H_17_N_3_OS (I), and 2-[(2-chloro-6-fluoro­benz­yl)sulfan­yl]-5-[4-(di­methyl­amino)­phen­yl]-1,3,4-oxa­diazole, C_17_H_15_ClFN_3_OS (II), were 96 and 92%, respectively. In the crystal structures of (I) and (II), C–H⋯π inter­actions are observed between neighboring mol­ecules. Hirshfeld surface analysis indicates that H⋯H and H⋯C/C⋯H inter­actions make the most important contributions to the crystal packing.

## Abstract 2

The title mol­ecules were synthesized by alkyl­ation of 5-[(4-di­methyl­amino)­phen­yl]-1,3,4-oxa­diazole-2-thiol. In the crystals, C–H⋯π inter­actions are observed between neighboring mol­ecules. Hirshfeld surface analysis indicates that H⋯H and H⋯C/C⋯H inter­actions make the most important contributions to the crystal packing.

## 1. Chemical context

For the synthesis of pharmacologically active heterocyclic compounds, a study of the relationship between structure and activity is of great inter­est. The various five-membered aromatic heterocyclic compounds have a diverse range of action. These include oxa­diazo­les, consisting of two carbon atoms, two nitro­gen atoms and one oxygen atom, which have four different isomeric structures: 1,2,3-oxa­diazole, 1,2,4-oxa­diazole, 1,2,5-oxa­diazole, 1,3,4-oxa­diazole.

There is much information in the literature indicating that 1,3,4-oxa­diazole compounds or substituted 1,3,4-oxa­diazo­les have a wide spectrum of biological activity (Şahin *et al.*, 2002 ▸; Erensoy *et al.*, 2020 ▸; Glomb & Świątek, 2021 ▸) with substituted 5-aryl-1,3,4-oxa­diazole-2(3*H*)thio­nes exhibiting anti-inflammatory, anti-cancer, analgesic and anti­convulsant activity (Chen *et al.*, 2007 ▸; Zheng *et al.*, 2010 ▸; Mamatha *et al.*, 2019 ▸; Pathak *et al.*, 2020 ▸). In this article, we report the synthesis and structure of two *S*-derivatives of 5-aryl-1,3,4-oxa­diazole-2-thiole derivatives. From the reaction of 5-[4-(di­methyl­amino)­phen­yl]-1,3,4-oxa­diazole-2-thiole with benzyl chloride or 2-chloro-6-fluoro­benzyl chloride, the corresponding *S*-products, 2-(benzyl­sulfan­yl)-5-[4-(di­methyl­amino)­phen­yl]-1,3,4-oxa­diazole (I) and 2-[(2-chloro-6-fluoro­benz­yl)sulfan­yl]-5-[4-(di­methyl­amino)­phen­yl]-1,3,4-oxa­diazole (II) were obtained in high yield. 
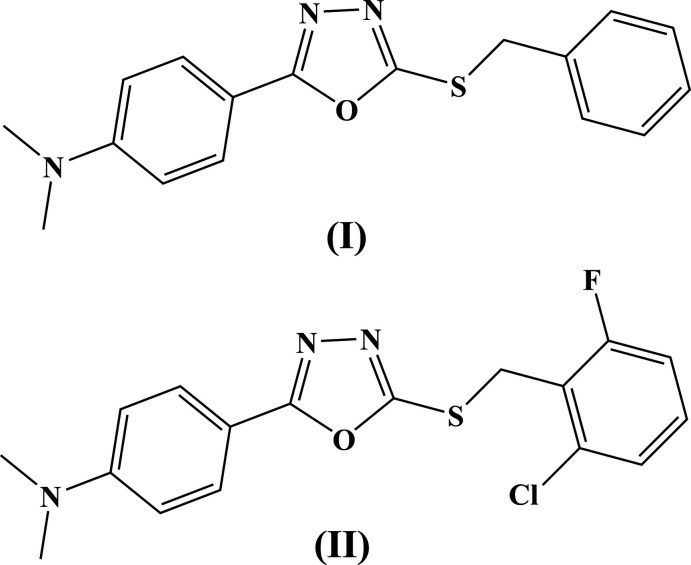


## 2. Structural commentary

Compound (I) crystallizes in space group *Ia*. The crystal studied was refined as an inversion twin with matrix [ 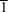 0 0, 0 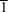 0, 0 0 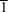 ] ; the resulting BASF value is 0.43 (2). Compound (II) crystallizes in *P*21_1_/*c*.

**Figure 1:**
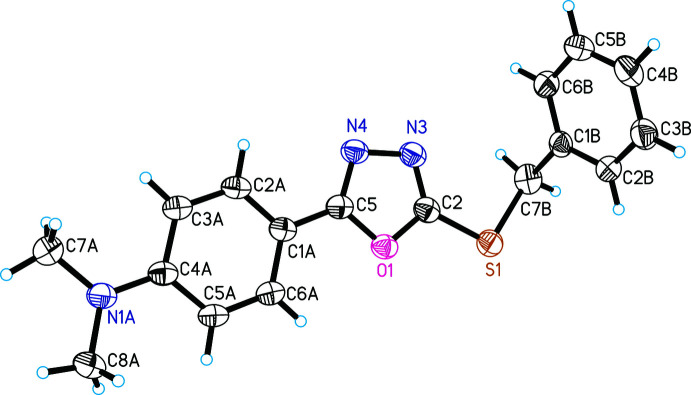
The asymmetric unit of (I) with atom labeling. Ellipsoids represent 30% probability levels.

In compounds (I) and (II), the oxa­diazole rings (centroid *Cg*1) are almost coplanar with the attached benzene (C1*A*–C6*A*, centroid *Cg*2) rings, forming dihedral angles of 3.36 (18) and 2.93 (14)°, respectively (Figs. 1 ▸ and 2 ▸). Such an arrangement of the benzene or phenyl fragment is also observed in many similar structures (Singh *et al.*, 2007 ▸; Zareef *et al.*, 2008 ▸; Zheng *et al.*, 2010 ▸; Ji & Xu 2011 ▸; Zou *et al.*, 2020 ▸). This arrangement indicates conjugation of π-electrons between the benzene and the 1,3,4-oxa­diazole rings.

The bond angle C2—S1—C7*B* is 99.79 (16)° in (I) and 100.11 (10)° in (II). The dihedral angle subtended by the benzene (C1*B*–C6*B*, centroid *Cg*3) and 1,3,4-oxa­diazole rings is 74.94 (10)° in (I) and 73.12 (7)°in (II).

## 3. Supra­molecular features

**Figure 3:**
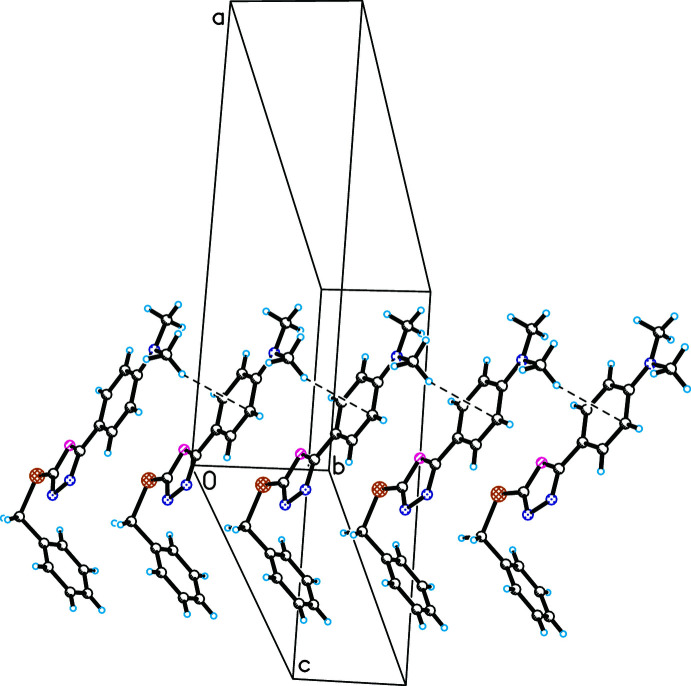
Observed weak inter­molecular C7*A*—H7*AC*⋯*Cg*2 inter­actions in the crystal structure of (I) (the mol­ecules are linked along the *b-*axis direction).

**Figure 4:**
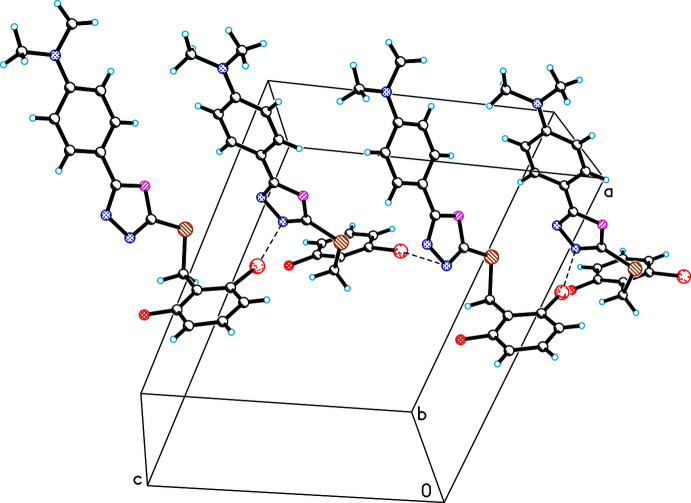
Observed inter­molecular C2*B*—Cl1⋯*Cg*1 inter­actions in the crystal structure of (II) (the mol­ecules are linked along the *c-*axis direction).

In crystal structures of the title compounds, weak inter­molecular contacts of the C—*X*⋯π type are observed. In (I), weak C7*A*—H7*AC*⋯*Cg*2 inter­actions link the mol­ecules, forming infinite chains along the *b-*axis direction (Fig. 3 ▸). Between these chains, other inter­actions of the C7*B*—H7*BA*⋯*Cg*3 type are observed, which consolidate the crystal structure (Table 1 ▸). In the crystal structure of (II), the formation of an infinite chain is also observed as a result of the C2*B*—Cl1⋯*Cg*1 inter­action, which links mol­ecules along the *c*-axis direction (Fig. 4 ▸). Inter­molecular C8*A*—H8*AB*⋯*Cg*3 and C7*B*—H7*BA*⋯*Cg*3 inter­actions between these chains consolidate the crystal structure (Table 2 ▸).

**Figure 5:**
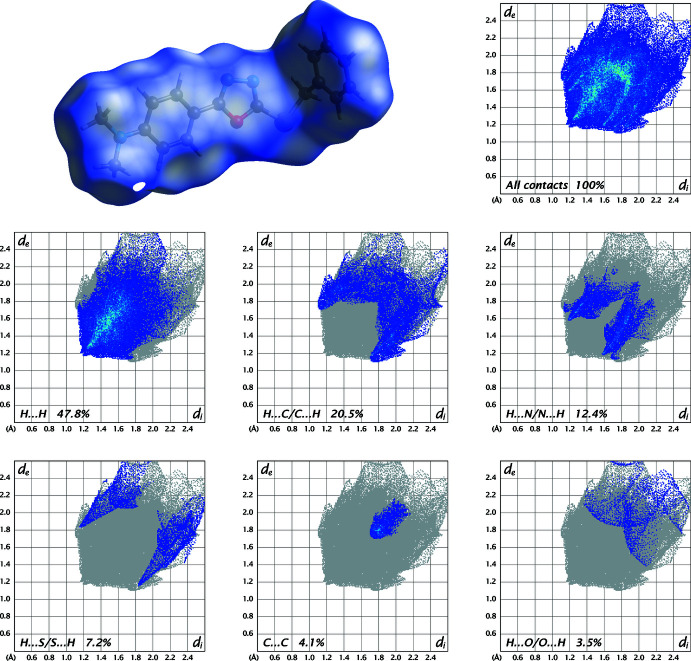
Three-dimensional Hirshfeld surfaces of compound (I) plotted over *d*_norm_ in the range 0.0145 to 1.3066 a.u. Hirshfeld fingerprint plots for all contacts and decomposed into H⋯H, H⋯C/C⋯H, H⋯N/N⋯H, H⋯S/S⋯H, C⋯C and H⋯O/O⋯H contacts. *d*_i_ and *d*_e_ denote the closest inter­nal and external distances (in Å) from a point on the surface.

In order to visualize and qu­antify the inter­molecular inter­actions in (I) and (II), a Hirshfeld surface analysis (Spackman & Jayatilaka, 2009 ▸) was performed with *Crystal Explorer 21* (Spackman *et al.*, 2021 ▸) and the associated two-dimensional fingerprint plots (McKinnon *et al.*, 2007 ▸) generated. The Hirshfeld surfaces for the mol­ecules in (I) and (II) are shown in Figs. 5 ▸ and 6 ▸ in which the two-dimensional fingerprint plots of the most dominant contacts are also presented.

For structure (I), H⋯H contacts are responsible for the largest contribution (47.8%) to the Hirshfeld surface. Besides these contacts, H⋯C/C⋯H (20.5%), H⋯N/N⋯H (12.4%), H⋯S/S⋯H (7.2%), C⋯C (4.1%) and H⋯O/O⋯H (3.5%) inter­actions contribute significantly to the total Hirshfeld surface (Fig. 5 ▸). The contributions of other contacts are O⋯C/C⋯O (2.0%), O⋯S/S⋯O (1.3%), S⋯C/C⋯S (0.9%), N⋯C/C⋯N (0.4%) and N⋯N (0.1%).

**Figure 6:**
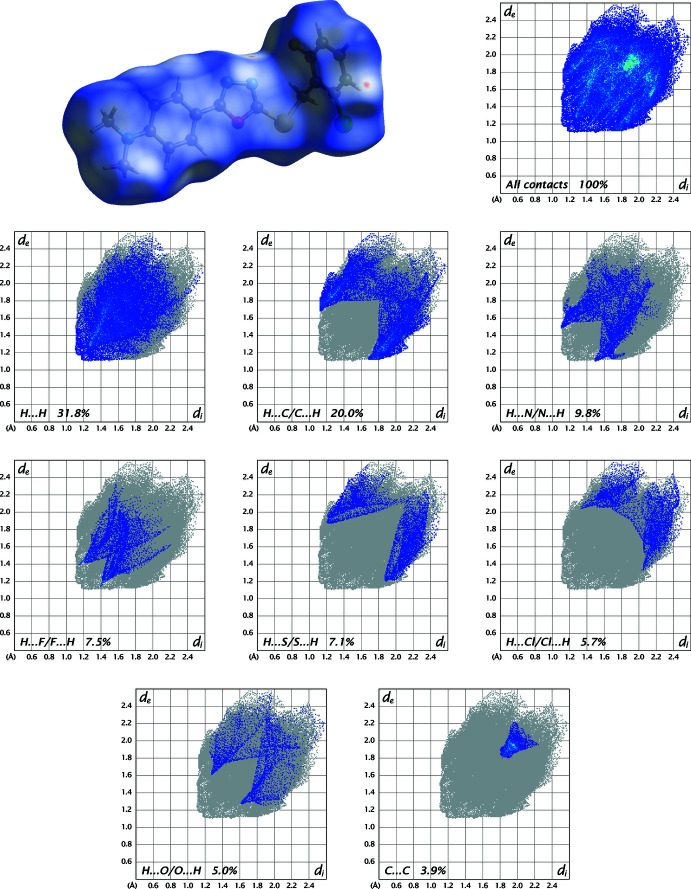
Three-dimensional Hirshfeld surfaces of the compound (II) plotted over *d*_norm_ in the range −0.0964 to 1.2943 a.u. Hirshfeld fingerprint plots for all contacts and decomposed into H⋯H, H⋯C/C⋯H, H⋯N/N⋯H, H⋯F/F⋯H, H⋯S/S⋯H, H⋯Cl/Cl⋯H, H⋯O/O⋯H and C⋯C contacts. *d*_i_ and *d*_e_ denote the closest inter­nal and external distances (in Å) from a point on the surface.

In the structure of (II), the percentage contributions of the most significant contacts differ because of the presence of H⋯F/F⋯H and H⋯Cl/Cl⋯H inter­actions and amount to H⋯H (31.8%), H⋯C/C⋯H (20.0%), H⋯N/N⋯H (9.8%), H⋯F/F⋯H (7.5%), H⋯S/S⋯H (7.1%), H⋯Cl/Cl⋯H (5.7%), H⋯O/O⋯H (5.0%) and C⋯C (3.9%) (Fig. 6 ▸). The contributions of other contacts are Cl⋯C/C⋯Cl (2.8%), Cl⋯F/F⋯Cl (1.4%), N⋯S/S⋯N (1.0%), Cl⋯O/O⋯Cl (0.9%), O⋯C/C⋯O (0.4%), N⋯C/C⋯N (0.4%), S⋯Cl/Cl⋯S (0.3%), S⋯C/C⋯S (0.3%) and N⋯O/O⋯N (0.2%).

As seen from Figs. 5 ▸ and 6 ▸, the most significant contributions to the overall Hirshfeld surface in the crystal structures of (I) and (II) are from H⋯H and H⋯C/C⋯H contacts (together they amount to more than 50% for both cases).

## 4. Database survey

A search in the Cambridge Structural Database (CSD, version 2022.3.0; Groom *et al.*, 2016 ▸) yielded 45 derivatives of 5-phenyl-1,3,4-oxa­diazole-2-thiole, nine of which are 2-(benz­yl­sulfan­yl)-5-phenyl-1,3,4-oxa­diazole derivatives, and no structure was found for a 5-[4-(di­methyl­amino)­phen­yl]-1,3,4-oxa­diazole-2-thiole derivative. When searching for similar structures in the CSD, two matches were found: 2-(4-meth­oxy­phen­yl)-5-({[3-(tri­fluoro­meth­yl)phen­yl] meth­yl}sulfan­yl)-1,3,4-oxa­diazole (SOXGOE; Hamdani *et al.*, 2020 ▸) and 2-benzyl­sulfanyl-5-(3,4,5-tri­meth­oxy­phen­yl)-1,3,4-oxa­diazole (GIDKEK; Chen *et al.*, 2007 ▸), in which the benzene rings and 1,3,4-oxa­diazole fragments are arranged in a similar manner as the title compounds. However, in the structures of SOXGOE and GIGKEK, inter­molecular inter­actions are not observed, the mol­ecules being stabilized mainly by van der Waals forces.

## 5. Synthesis and crystallization

A mixture of 5-[4-(di­methyl­amino)­phen­yl]-1,3,4-oxa­diazole-2-thiole (0.005 mol), benzyl chloride or 2-chloro-6-fluoro­benzyl chloride (0.005 mol) and K_2_CO_3_ (0.005 mol) was boiled in 20 ml of dry acetone for 6 h. The solvent was then removed, the residue washed with water and with 2% NaOH solution to remove unreacted oxa­diazo­lthione, and then washed with water until neutral. The resulting target products were dried in air and recrystallized from ethanol solution. Compound (I): yield 96%, m.p. 404–405 K. Compound (II): yield 92%, m.p. 406–407 K.

## 6. Refinement

Crystal data, data collection and structure refinement details are summarized in Table 3 ▸. H atoms were positioned geom­etrically (with C—H distances of 0.97 Å for CH_2_, 0.96 Å for CH_3_ and 0.93 Å for C_ar_) and included in the refinement in a riding-motion approximation with *U*_iso_(H) = 1.2*U*_eq_(C) [*U*_iso_ = 1.5*U*_eq_(C) for methyl H atoms]. For (I), the crystal studied was refined as an inversion twin with matrix [ 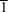 0 0, 0 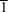 0, 0 0 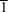 ] ; the resulting BASF value is 0.43 (2).

## Supplementary Material

Crystal structure: contains datablock(s) I, II, Global. DOI: 10.1107/S2056989023004164/dx2051sup1.cif

Structure factors: contains datablock(s) I. DOI: 10.1107/S2056989023004164/dx2051Isup2.hkl

Structure factors: contains datablock(s) II. DOI: 10.1107/S2056989023004164/dx2051IIsup3.hkl

Supporting information file. DOI: 10.1107/S2056989023004164/dx2051Isup4.cml

Supporting information file. DOI: 10.1107/S2056989023004164/dx2051IIsup5.cml

CCDC references: 2262491, 2262490

Additional supporting information:  crystallographic information; 3D view; checkCIF report

## Figures and Tables

**Figure 2:**
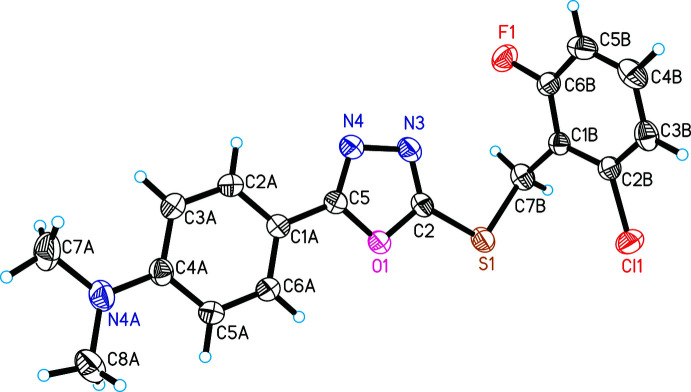
The asymmetric unit of (II) with atom labeling. Ellipsoids represent 30% probability levels.

**Table 1:** Hydrogen-bond geometry (Å, °) for (I) *Cg*2 and *Cg*3 are the centroids of the C1*A*–C6*A* and C1*B*–C6*B* rings, respectively.

*D*—H⋯*A*	*D*—H	H⋯*A*	*D*⋯*A*	*D*—H⋯*A*
C7*A*—H7*AC*⋯*Cg*2^i^	0.96	2.80	3.626 (4)	145
C7*B*—H7*BA*⋯*Cg*3^ii^	0.97	2.93	3.738 (4)	141

Symmetry codes: (i) 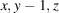 ; (ii) 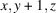 .

**Table 2:** Hydrogen-bond geometry (Å, °) for (II) *Cg*1 and *Cg*3 are the centroids of the O1/C2/N3/N4/C5 and C1*B*–C6*B* rings, respectively.

*D*—H⋯*A*	*D*—H	H⋯*A*	*D*⋯*A*	*D*—H⋯*A*
C2*B*—Cl1⋯*Cg*1^i^	1.74 (1)	3.30 (1)	4.939 (2)	156 (1)
C8*A*—H8*AB*⋯*Cg*3^ii^	0.96	2.94	3.857 (3)	161
C7*B*—H7*BA*⋯*Cg*3^iii^	0.97	2.85	3.674 (2)	143

Symmetry codes: (i) 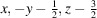 ; (ii) 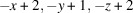 ; (iii) 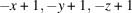 .

**Table 3:** Experimental details

	(I)	(II)
Crystal data		
Chemical formula	C_17_H_17_N_3_OS	C_17_H_15_ClFN_3_OS
*M*_r_	311.39	363.83
Crystal system, space group	Monoclinic, *I* *a*	Monoclinic, *P*2_1_/*c*
Temperature (K)	297	296
*a*, *b*, *c* (Å)	16.816 (3), 4.7848 (10), 20.123 (4)	16.308 (3), 7.9787 (16), 13.072 (3)
β (°)	105.96 (3)	103.33 (3)
*V* (Å^3^)	1556.7 (6)	1655.1 (6)
*Z*	4	4
Radiation type	Cu *K*α	Cu *K*α
μ (mm^−1^)	1.88	3.40
Crystal size (mm)	0.35 × 0.20 × 0.15	0.30 × 0.25 × 0.15

Data collection		
Diffractometer	XtaLAB Synergy, Single source at home/near, HyPix3000	XtaLAB Synergy, Single source at home/near, HyPix3000
Absorption correction	Multi-scan (*SADABS*; Krause *et al.*, 2015 ▸)	Multi-scan (*SADABS*; Krause *et al.*, 2015 ▸)
*T*_min_, *T*_max_	0.749, 1.000	0.704, 1.000
No. of measured, independent and observed [*I* > 2σ(*I*)] reflections	6572, 2732, 2583	8579, 3181, 2771
*R*_int_	0.026	0.021
(sin θ/λ)_max_ (Å^−1^)	0.615	0.615

Refinement		
*R*[*F* ^2^ > 2σ(*F* ^2^)], *wR*(*F* ^2^), *S*	0.032, 0.089, 1.04	0.039, 0.106, 1.05
No. of reflections	2732	3181
No. of parameters	202	219
No. of restraints	2	0
H-atom treatment	H-atom parameters constrained	H-atom parameters constrained
Δρ_max_, Δρ_min_ (e Å^−3^)	0.15, −0.21	0.18, −0.33
Absolute structure	Refined as an inversion twin	–
Absolute structure parameter	0.43 (2)	–

Computer programs: *CrysAlis PRO* (Rigaku OD, 2021 ▸), *SHELXS97*, *SHELXTL* (Sheldrick, 2015 ▸) and *XP* in *SHELXTL* (Sheldrick, 2008 ▸), *PLATON* (Spek, 2020 ▸) and *publCIF* (Westrip, 2010 ▸).

## supplementary crystallographic information

### 2-(Benzylsulfanyl)-5-[4-(dimethylamino)phenyl]-1,3,4-oxadiazole (I) Crystal data

**Table:**

C_17_H_17_N_3_OS	*F*(000) = 656
*M**_r_* = 311.39	*D*_x_ = 1.329 Mg m^−^^3^
Monoclinic, *I**a*	Cu *K*α radiation, λ = 1.54184 Å
*a* = 16.816 (3) Å	Cell parameters from 4218 reflections
*b* = 4.7848 (10) Å	θ = 3.0–71.2°
*c* = 20.123 (4) Å	µ = 1.88 mm^−^^1^
β = 105.96 (3)°	*T* = 297 K
*V* = 1556.7 (6) Å^3^	Prizmatic, colorless
*Z* = 4	0.35 × 0.20 × 0.15 mm

### 2-(Benzylsulfanyl)-5-[4-(dimethylamino)phenyl]-1,3,4-oxadiazole (I) Data collection

**Table:**

XtaLAB Synergy, Single source at home/near, HyPix3000 diffractometer	2732 independent reflections
Radiation source: micro-focus sealed X-ray tube, PhotonJet (Cu) X-ray Source	2583 reflections with *I* > 2σ(*I*)
Detector resolution: 10.0000 pixels mm^-1^	*R*_int_ = 0.026
ω scans	θ_max_ = 71.5°, θ_min_ = 4.6°
Absorption correction: multi-scan (SADABS; Krause *et al.*, 2015)	*h* = −20→20
*T*_min_ = 0.749, *T*_max_ = 1.000	*k* = −5→5
6572 measured reflections	*l* = −24→24

### 2-(Benzylsulfanyl)-5-[4-(dimethylamino)phenyl]-1,3,4-oxadiazole (I) Refinement

**Table:**

Refinement on *F*^2^	Hydrogen site location: inferred from neighbouring sites
Least-squares matrix: full	H-atom parameters constrained
*R*[*F*^2^ > 2σ(*F*^2^)] = 0.032	*w* = 1/[σ^2^(*F*_o_^2^) + (0.0541*P*)^2^] where *P* = (*F*_o_^2^ + 2*F*_c_^2^)/3
*wR*(*F*^2^) = 0.089	(Δ/σ)_max_ < 0.001
*S* = 1.04	Δρ_max_ = 0.15 e Å^−^^3^
2732 reflections	Δρ_min_ = −0.21 e Å^−^^3^
202 parameters	Absolute structure: Refined as an inversion twin
2 restraints	Absolute structure parameter: 0.43 (2)
Primary atom site location: dual	

### 2-(Benzylsulfanyl)-5-[4-(dimethylamino)phenyl]-1,3,4-oxadiazole (I) Special details

**Table:**

Geometry. All esds (except the esd in the dihedral angle between two l.s. planes) are estimated using the full covariance matrix. The cell esds are taken into account individually in the estimation of esds in distances, angles and torsion angles; correlations between esds in cell parameters are only used when they are defined by crystal symmetry. An approximate (isotropic) treatment of cell esds is used for estimating esds involving l.s. planes.
Refinement. Refined as a 2-component inversion twin.

### 2-(Benzylsulfanyl)-5-[4-(dimethylamino)phenyl]-1,3,4-oxadiazole (I) Fractional atomic coordinates and isotropic or equivalent isotropic displacement parameters (Å^2^)

**Table:**

	*x*	*y*	*z*	*U*_iso_*/*U*_eq_	
S1	0.35188 (5)	0.65967 (15)	0.24394 (4)	0.0675 (2)	
O1	0.38557 (12)	0.2829 (4)	0.15990 (10)	0.0546 (4)	
N3	0.25142 (15)	0.3621 (5)	0.13720 (14)	0.0607 (6)	
N4	0.26466 (15)	0.1612 (5)	0.09008 (14)	0.0595 (6)	
N1A	0.51675 (14)	−0.6015 (5)	−0.02778 (13)	0.0614 (6)	
C2	0.32356 (17)	0.4249 (6)	0.17577 (14)	0.0561 (6)	
C5	0.34386 (17)	0.1194 (6)	0.10548 (14)	0.0518 (6)	
C1A	0.38914 (15)	−0.0700 (6)	0.07351 (13)	0.0497 (5)	
C2A	0.34682 (16)	−0.2469 (6)	0.02095 (15)	0.0541 (6)	
H2AA	0.289305	−0.245654	0.007619	0.065*	
C3A	0.38831 (17)	−0.4238 (6)	−0.01174 (14)	0.0543 (6)	
H3AA	0.358087	−0.539335	−0.046814	0.065*	
C4A	0.47522 (16)	−0.4349 (6)	0.00637 (14)	0.0514 (6)	
C5A	0.51775 (17)	−0.2603 (6)	0.06167 (16)	0.0555 (6)	
H5AA	0.575192	−0.266824	0.076730	0.067*	
C6A	0.47585 (16)	−0.0824 (6)	0.09332 (14)	0.0549 (6)	
H6AA	0.505497	0.032849	0.128737	0.066*	
C7A	0.4722 (2)	−0.7707 (7)	−0.08530 (18)	0.0681 (8)	
H7AA	0.510695	−0.863023	−0.105354	0.102*	
H7AB	0.436148	−0.653916	−0.119410	0.102*	
H7AC	0.439939	−0.907932	−0.069428	0.102*	
C8A	0.60506 (19)	−0.6514 (7)	0.0000 (2)	0.0718 (9)	
H8AA	0.622912	−0.784539	−0.028621	0.108*	
H8AB	0.615965	−0.723417	0.046118	0.108*	
H8AC	0.634547	−0.479131	0.000665	0.108*	
C1B	0.20329 (17)	0.5859 (6)	0.27839 (14)	0.0536 (6)	
C2B	0.23312 (18)	0.5105 (7)	0.34681 (15)	0.0605 (7)	
H2BA	0.283977	0.579459	0.372557	0.073*	
C3B	0.1890 (2)	0.3346 (7)	0.37792 (18)	0.0723 (9)	
H3BA	0.209881	0.285927	0.424205	0.087*	
C4B	0.1137 (3)	0.2317 (9)	0.3397 (2)	0.0826 (10)	
H4BA	0.083364	0.114435	0.360365	0.099*	
C5B	0.0837 (2)	0.3010 (10)	0.2720 (2)	0.0863 (11)	
H5BA	0.033294	0.228498	0.246285	0.104*	
C6B	0.1276 (2)	0.4787 (8)	0.24093 (17)	0.0710 (8)	
H6BA	0.106285	0.526586	0.194651	0.085*	
C7B	0.2500 (2)	0.7858 (6)	0.24513 (19)	0.0687 (8)	
H7BA	0.256392	0.962089	0.269826	0.082*	
H7BB	0.217555	0.820846	0.197995	0.082*	

### 2-(Benzylsulfanyl)-5-[4-(dimethylamino)phenyl]-1,3,4-oxadiazole (I) Atomic displacement parameters (Å^2^)

**Table:**

	*U*^11^	*U*^22^	*U*^33^	*U*^12^	*U*^13^	*U*^23^
S1	0.0666 (4)	0.0746 (4)	0.0645 (4)	−0.0124 (4)	0.0234 (3)	−0.0030 (4)
O1	0.0501 (10)	0.0581 (9)	0.0555 (10)	−0.0021 (8)	0.0144 (8)	0.0069 (8)
N3	0.0516 (12)	0.0674 (14)	0.0641 (14)	0.0038 (10)	0.0177 (11)	0.0037 (11)
N4	0.0465 (12)	0.0681 (14)	0.0619 (14)	0.0035 (10)	0.0116 (10)	0.0036 (11)
N1A	0.0471 (12)	0.0666 (14)	0.0680 (15)	−0.0002 (10)	0.0114 (11)	−0.0029 (11)
C2	0.0564 (16)	0.0568 (14)	0.0578 (15)	−0.0004 (11)	0.0201 (13)	0.0116 (12)
C5	0.0471 (13)	0.0552 (13)	0.0515 (14)	−0.0025 (11)	0.0108 (11)	0.0121 (11)
C1A	0.0448 (12)	0.0528 (12)	0.0506 (13)	0.0000 (10)	0.0115 (11)	0.0127 (10)
C2A	0.0398 (13)	0.0597 (14)	0.0601 (15)	−0.0042 (10)	0.0092 (11)	0.0115 (12)
C3A	0.0451 (13)	0.0565 (14)	0.0571 (15)	−0.0047 (11)	0.0069 (11)	0.0061 (12)
C4A	0.0438 (12)	0.0521 (13)	0.0556 (15)	−0.0040 (10)	0.0091 (11)	0.0119 (11)
C5A	0.0400 (12)	0.0612 (14)	0.0600 (15)	−0.0034 (11)	0.0048 (12)	0.0073 (12)
C6A	0.0469 (13)	0.0576 (14)	0.0552 (14)	−0.0041 (11)	0.0058 (11)	0.0041 (12)
C7A	0.0636 (18)	0.0708 (18)	0.0676 (19)	−0.0046 (15)	0.0145 (15)	−0.0064 (15)
C8A	0.0473 (15)	0.084 (2)	0.084 (2)	0.0032 (15)	0.0178 (15)	−0.0042 (17)
C1B	0.0555 (14)	0.0531 (13)	0.0541 (14)	0.0088 (11)	0.0184 (12)	−0.0046 (11)
C2B	0.0550 (15)	0.0725 (17)	0.0527 (15)	−0.0006 (13)	0.0126 (12)	−0.0028 (12)
C3B	0.071 (2)	0.089 (2)	0.0609 (18)	0.0056 (16)	0.0252 (16)	0.0073 (15)
C4B	0.074 (2)	0.101 (3)	0.084 (2)	−0.0115 (19)	0.042 (2)	−0.006 (2)
C5B	0.0556 (18)	0.119 (3)	0.087 (2)	−0.0168 (18)	0.0245 (17)	−0.027 (2)
C6B	0.0580 (16)	0.097 (2)	0.0563 (17)	0.0077 (16)	0.0121 (14)	−0.0097 (16)
C7B	0.083 (2)	0.0529 (15)	0.0729 (19)	0.0070 (14)	0.0255 (16)	0.0060 (13)

### 2-(Benzylsulfanyl)-5-[4-(dimethylamino)phenyl]-1,3,4-oxadiazole (I) Geometric parameters (Å, º)

**Table:**

S1—C2	1.735 (3)	C7A—H7AA	0.9600
S1—C7B	1.823 (4)	C7A—H7AB	0.9600
O1—C2	1.354 (3)	C7A—H7AC	0.9600
O1—C5	1.371 (3)	C8A—H8AA	0.9600
N3—C2	1.283 (4)	C8A—H8AB	0.9600
N3—N4	1.410 (4)	C8A—H8AC	0.9600
N4—C5	1.297 (4)	C1B—C2B	1.378 (4)
N1A—C4A	1.364 (4)	C1B—C6B	1.387 (5)
N1A—C7A	1.442 (4)	C1B—C7B	1.506 (5)
N1A—C8A	1.455 (4)	C2B—C3B	1.381 (5)
C5—C1A	1.444 (4)	C2B—H2BA	0.9300
C1A—C2A	1.388 (4)	C3B—C4B	1.379 (6)
C1A—C6A	1.403 (4)	C3B—H3BA	0.9300
C2A—C3A	1.374 (4)	C4B—C5B	1.358 (7)
C2A—H2AA	0.9300	C4B—H4BA	0.9300
C3A—C4A	1.407 (4)	C5B—C6B	1.383 (6)
C3A—H3AA	0.9300	C5B—H5BA	0.9300
C4A—C5A	1.418 (4)	C6B—H6BA	0.9300
C5A—C6A	1.369 (4)	C7B—H7BA	0.9700
C5A—H5AA	0.9300	C7B—H7BB	0.9700
C6A—H6AA	0.9300		
			
C2—S1—C7B	99.79 (16)	N1A—C7A—H7AC	109.5
C2—O1—C5	102.5 (2)	H7AA—C7A—H7AC	109.5
C2—N3—N4	105.6 (2)	H7AB—C7A—H7AC	109.5
C5—N4—N3	106.6 (2)	N1A—C8A—H8AA	109.5
C4A—N1A—C7A	120.5 (2)	N1A—C8A—H8AB	109.5
C4A—N1A—C8A	120.8 (3)	H8AA—C8A—H8AB	109.5
C7A—N1A—C8A	117.8 (3)	N1A—C8A—H8AC	109.5
N3—C2—O1	113.6 (3)	H8AA—C8A—H8AC	109.5
N3—C2—S1	129.6 (2)	H8AB—C8A—H8AC	109.5
O1—C2—S1	116.7 (2)	C2B—C1B—C6B	118.3 (3)
N4—C5—O1	111.6 (3)	C2B—C1B—C7B	121.3 (3)
N4—C5—C1A	128.5 (3)	C6B—C1B—C7B	120.4 (3)
O1—C5—C1A	119.8 (2)	C1B—C2B—C3B	121.3 (3)
C2A—C1A—C6A	117.7 (3)	C1B—C2B—H2BA	119.3
C2A—C1A—C5	120.0 (2)	C3B—C2B—H2BA	119.3
C6A—C1A—C5	122.3 (2)	C4B—C3B—C2B	119.3 (3)
C3A—C2A—C1A	121.3 (3)	C4B—C3B—H3BA	120.3
C3A—C2A—H2AA	119.4	C2B—C3B—H3BA	120.3
C1A—C2A—H2AA	119.4	C5B—C4B—C3B	120.2 (4)
C2A—C3A—C4A	121.8 (3)	C5B—C4B—H4BA	119.9
C2A—C3A—H3AA	119.1	C3B—C4B—H4BA	119.9
C4A—C3A—H3AA	119.1	C4B—C5B—C6B	120.4 (4)
N1A—C4A—C3A	122.1 (3)	C4B—C5B—H5BA	119.8
N1A—C4A—C5A	121.5 (2)	C6B—C5B—H5BA	119.8
C3A—C4A—C5A	116.4 (3)	C5B—C6B—C1B	120.4 (3)
C6A—C5A—C4A	121.2 (3)	C5B—C6B—H6BA	119.8
C6A—C5A—H5AA	119.4	C1B—C6B—H6BA	119.8
C4A—C5A—H5AA	119.4	C1B—C7B—S1	113.7 (2)
C5A—C6A—C1A	121.4 (3)	C1B—C7B—H7BA	108.8
C5A—C6A—H6AA	119.3	S1—C7B—H7BA	108.8
C1A—C6A—H6AA	119.3	C1B—C7B—H7BB	108.8
N1A—C7A—H7AA	109.5	S1—C7B—H7BB	108.8
N1A—C7A—H7AB	109.5	H7BA—C7B—H7BB	107.7
H7AA—C7A—H7AB	109.5		
			
C2—N3—N4—C5	0.8 (3)	C7A—N1A—C4A—C5A	−178.0 (3)
N4—N3—C2—O1	−0.6 (3)	C8A—N1A—C4A—C5A	13.0 (4)
N4—N3—C2—S1	179.7 (2)	C2A—C3A—C4A—N1A	−177.2 (3)
C5—O1—C2—N3	0.2 (3)	C2A—C3A—C4A—C5A	2.0 (4)
C5—O1—C2—S1	179.95 (18)	N1A—C4A—C5A—C6A	176.4 (3)
C7B—S1—C2—N3	0.7 (3)	C3A—C4A—C5A—C6A	−2.8 (4)
C7B—S1—C2—O1	−179.1 (2)	C4A—C5A—C6A—C1A	1.5 (4)
N3—N4—C5—O1	−0.8 (3)	C2A—C1A—C6A—C5A	0.6 (4)
N3—N4—C5—C1A	178.9 (2)	C5—C1A—C6A—C5A	−179.0 (3)
C2—O1—C5—N4	0.4 (3)	C6B—C1B—C2B—C3B	−0.5 (4)
C2—O1—C5—C1A	−179.3 (2)	C7B—C1B—C2B—C3B	177.8 (3)
N4—C5—C1A—C2A	−3.2 (4)	C1B—C2B—C3B—C4B	0.2 (5)
O1—C5—C1A—C2A	176.4 (2)	C2B—C3B—C4B—C5B	0.6 (6)
N4—C5—C1A—C6A	176.4 (3)	C3B—C4B—C5B—C6B	−1.0 (6)
O1—C5—C1A—C6A	−4.0 (4)	C4B—C5B—C6B—C1B	0.7 (6)
C6A—C1A—C2A—C3A	−1.5 (4)	C2B—C1B—C6B—C5B	0.1 (5)
C5—C1A—C2A—C3A	178.2 (2)	C7B—C1B—C6B—C5B	−178.3 (3)
C1A—C2A—C3A—C4A	0.1 (4)	C2B—C1B—C7B—S1	61.9 (3)
C7A—N1A—C4A—C3A	1.1 (4)	C6B—C1B—C7B—S1	−119.8 (3)
C8A—N1A—C4A—C3A	−167.9 (3)	C2—S1—C7B—C1B	77.7 (3)

### 2-(Benzylsulfanyl)-5-[4-(dimethylamino)phenyl]-1,3,4-oxadiazole (I) Hydrogen-bond geometry (Å, º)

*Cg*2 and *Cg*3 are the centroids of the C1*A*–C6*A* and C1*B*–C6*B* rings, respectively.

**Table:**

*D*—H···*A*	*D*—H	H···*A*	*D*···*A*	*D*—H···*A*
C7*A*—H7*AC*···*Cg*2^i^	0.96	2.80	3.626 (4)	145
C7*B*—H7*BA*···*Cg*3^ii^	0.97	2.93	3.738 (4)	141

Symmetry codes: (i) *x*, *y*−1, *z*; (ii) *x*, *y*+1, *z*.

### 2-[(2-Chloro-6-fluorobenzyl)sulfanyl]-5-[4-(dimethylamino)phenyl]-1,3,4-oxadiazole (II) Crystal data

**Table:**

C_17_H_15_ClFN_3_OS	*F*(000) = 752
*M**_r_* = 363.83	*D*_x_ = 1.460 Mg m^−^^3^
Monoclinic, *P*2_1_/*c*	Cu *K*α radiation, λ = 1.54184 Å
*a* = 16.308 (3) Å	Cell parameters from 4884 reflections
*b* = 7.9787 (16) Å	θ = 2.8–70.7°
*c* = 13.072 (3) Å	µ = 3.40 mm^−^^1^
β = 103.33 (3)°	*T* = 296 K
*V* = 1655.1 (6) Å^3^	Prizmatic, colorless
*Z* = 4	0.30 × 0.25 × 0.15 mm

### 2-[(2-Chloro-6-fluorobenzyl)sulfanyl]-5-[4-(dimethylamino)phenyl]-1,3,4-oxadiazole (II) Data collection

**Table:**

XtaLAB Synergy, Single source at home/near, HyPix3000 diffractometer	3181 independent reflections
Radiation source: micro-focus sealed X-ray tube, PhotonJet (Cu) X-ray Source	2771 reflections with *I* > 2σ(*I*)
Detector resolution: 10.0000 pixels mm^-1^	*R*_int_ = 0.021
ω scans	θ_max_ = 71.5°, θ_min_ = 2.8°
Absorption correction: multi-scan (SADABS; Krause *et al.*, 2015)	*h* = −19→19
*T*_min_ = 0.704, *T*_max_ = 1.000	*k* = −9→9
8579 measured reflections	*l* = −14→15

### 2-[(2-Chloro-6-fluorobenzyl)sulfanyl]-5-[4-(dimethylamino)phenyl]-1,3,4-oxadiazole (II) Refinement

**Table:**

Refinement on *F*^2^	Primary atom site location: dual
Least-squares matrix: full	Hydrogen site location: inferred from neighbouring sites
*R*[*F*^2^ > 2σ(*F*^2^)] = 0.039	H-atom parameters constrained
*wR*(*F*^2^) = 0.106	*w* = 1/[σ^2^(*F*_o_^2^) + (0.0548*P*)^2^ + 0.3561*P*] where *P* = (*F*_o_^2^ + 2*F*_c_^2^)/3
*S* = 1.05	(Δ/σ)_max_ = 0.001
3181 reflections	Δρ_max_ = 0.18 e Å^−^^3^
219 parameters	Δρ_min_ = −0.33 e Å^−^^3^
0 restraints	

### 2-[(2-Chloro-6-fluorobenzyl)sulfanyl]-5-[4-(dimethylamino)phenyl]-1,3,4-oxadiazole (II) Special details

**Table:**

Geometry. All esds (except the esd in the dihedral angle between two l.s. planes) are estimated using the full covariance matrix. The cell esds are taken into account individually in the estimation of esds in distances, angles and torsion angles; correlations between esds in cell parameters are only used when they are defined by crystal symmetry. An approximate (isotropic) treatment of cell esds is used for estimating esds involving l.s. planes.

### 2-[(2-Chloro-6-fluorobenzyl)sulfanyl]-5-[4-(dimethylamino)phenyl]-1,3,4-oxadiazole (II) Fractional atomic coordinates and isotropic or equivalent isotropic displacement parameters (Å^2^)

**Table:**

	*x*	*y*	*z*	*U*_iso_*/*U*_eq_	
S1	0.70024 (3)	0.25638 (6)	0.67262 (4)	0.05766 (17)	
F1	0.53784 (8)	0.62390 (19)	0.74016 (9)	0.0710 (4)	
Cl1	0.65329 (4)	0.42921 (8)	0.43372 (4)	0.07189 (19)	
N3	0.70438 (10)	0.4438 (2)	0.85013 (13)	0.0575 (4)	
N4	0.76430 (11)	0.4635 (2)	0.94693 (13)	0.0591 (4)	
N4A	1.13318 (11)	0.2937 (3)	1.24513 (14)	0.0645 (5)	
O1	0.81509 (8)	0.28552 (16)	0.84836 (9)	0.0484 (3)	
C2	0.73729 (11)	0.3394 (2)	0.79712 (14)	0.0469 (4)	
C5	0.82736 (11)	0.3695 (2)	0.94229 (13)	0.0459 (4)	
C1A	0.90528 (11)	0.3437 (2)	1.02033 (13)	0.0458 (4)	
C2A	0.91795 (13)	0.4247 (3)	1.11650 (15)	0.0569 (5)	
H2AA	0.875254	0.491431	1.130992	0.068*	
C3A	0.99218 (13)	0.4085 (3)	1.19079 (15)	0.0583 (5)	
H3AA	0.998800	0.464500	1.254560	0.070*	
C4A	1.05833 (11)	0.3090 (2)	1.17239 (14)	0.0490 (4)	
C5A	1.04385 (13)	0.2249 (3)	1.07595 (16)	0.0583 (5)	
H5AA	1.085423	0.154883	1.061691	0.070*	
C6A	0.96948 (13)	0.2437 (3)	1.00180 (15)	0.0560 (5)	
H6AA	0.962246	0.187883	0.937881	0.067*	
C7A	1.14655 (15)	0.3781 (4)	1.34498 (18)	0.0783 (7)	
H7AA	1.103965	0.344344	1.380639	0.118*	
H7AB	1.143475	0.497119	1.333810	0.118*	
H7AC	1.201117	0.349276	1.387036	0.118*	
C8A	1.20022 (14)	0.1914 (3)	1.2238 (2)	0.0734 (6)	
H8AA	1.180549	0.078396	1.209806	0.110*	
H8AB	1.247110	0.192586	1.283686	0.110*	
H8AC	1.217624	0.234985	1.163709	0.110*	
C1B	0.59678 (10)	0.5336 (2)	0.60183 (12)	0.0413 (4)	
C2B	0.62301 (10)	0.5823 (2)	0.51172 (13)	0.0447 (4)	
C3B	0.62432 (12)	0.7473 (3)	0.48082 (16)	0.0555 (5)	
H3BA	0.643266	0.775274	0.421154	0.067*	
C4B	0.59745 (14)	0.8693 (3)	0.53885 (19)	0.0641 (6)	
H4BA	0.598734	0.980957	0.518853	0.077*	
C5B	0.56857 (13)	0.8285 (3)	0.62650 (18)	0.0618 (5)	
H5BA	0.549420	0.911110	0.665483	0.074*	
C6B	0.56865 (11)	0.6628 (3)	0.65515 (14)	0.0487 (4)	
C7B	0.59662 (12)	0.3548 (2)	0.63726 (16)	0.0537 (5)	
H7BA	0.561130	0.289986	0.581406	0.064*	
H7BB	0.571488	0.350251	0.697611	0.064*	

### 2-[(2-Chloro-6-fluorobenzyl)sulfanyl]-5-[4-(dimethylamino)phenyl]-1,3,4-oxadiazole (II) Atomic displacement parameters (Å^2^)

**Table:**

	*U*^11^	*U*^22^	*U*^33^	*U*^12^	*U*^13^	*U*^23^
S1	0.0609 (3)	0.0489 (3)	0.0553 (3)	0.0077 (2)	−0.0030 (2)	−0.0039 (2)
F1	0.0611 (7)	0.1044 (10)	0.0511 (6)	0.0122 (7)	0.0199 (5)	−0.0031 (6)
Cl1	0.0721 (4)	0.0899 (4)	0.0532 (3)	0.0172 (3)	0.0134 (2)	−0.0173 (3)
N3	0.0510 (9)	0.0600 (10)	0.0562 (9)	0.0113 (8)	0.0010 (7)	0.0001 (8)
N4	0.0550 (9)	0.0651 (10)	0.0532 (9)	0.0145 (8)	0.0046 (7)	−0.0054 (8)
N4A	0.0483 (9)	0.0850 (12)	0.0551 (9)	0.0034 (9)	0.0013 (7)	0.0009 (9)
O1	0.0478 (7)	0.0486 (7)	0.0460 (6)	0.0071 (5)	0.0050 (5)	−0.0008 (5)
C2	0.0466 (9)	0.0408 (9)	0.0500 (9)	0.0025 (8)	0.0043 (8)	0.0053 (7)
C5	0.0481 (10)	0.0438 (9)	0.0455 (9)	0.0032 (8)	0.0102 (7)	0.0005 (7)
C1A	0.0454 (9)	0.0463 (9)	0.0447 (9)	0.0038 (8)	0.0088 (7)	0.0015 (7)
C2A	0.0552 (11)	0.0628 (12)	0.0518 (10)	0.0154 (9)	0.0107 (9)	−0.0077 (9)
C3A	0.0636 (12)	0.0639 (12)	0.0449 (10)	0.0076 (10)	0.0072 (9)	−0.0093 (9)
C4A	0.0459 (10)	0.0549 (10)	0.0454 (9)	−0.0020 (8)	0.0093 (7)	0.0066 (8)
C5A	0.0504 (11)	0.0717 (13)	0.0524 (10)	0.0171 (10)	0.0110 (9)	−0.0048 (9)
C6A	0.0560 (11)	0.0650 (12)	0.0455 (10)	0.0124 (9)	0.0087 (9)	−0.0080 (9)
C7A	0.0620 (13)	0.109 (2)	0.0565 (12)	−0.0168 (14)	−0.0021 (10)	−0.0042 (13)
C8A	0.0484 (11)	0.0855 (16)	0.0824 (15)	0.0075 (11)	0.0071 (10)	0.0184 (13)
C1B	0.0319 (8)	0.0469 (9)	0.0415 (8)	−0.0017 (7)	0.0008 (6)	−0.0021 (7)
C2B	0.0340 (8)	0.0545 (10)	0.0423 (8)	0.0003 (7)	0.0018 (7)	−0.0044 (7)
C3B	0.0431 (10)	0.0641 (12)	0.0553 (11)	−0.0049 (9)	0.0031 (8)	0.0125 (9)
C4B	0.0555 (12)	0.0470 (11)	0.0818 (15)	−0.0011 (9)	−0.0005 (10)	0.0053 (10)
C5B	0.0522 (11)	0.0545 (11)	0.0731 (13)	0.0080 (9)	0.0032 (10)	−0.0161 (10)
C6B	0.0367 (8)	0.0633 (11)	0.0437 (9)	0.0029 (8)	0.0048 (7)	−0.0064 (8)
C7B	0.0448 (10)	0.0522 (10)	0.0589 (11)	−0.0064 (8)	0.0014 (8)	0.0019 (8)

### 2-[(2-Chloro-6-fluorobenzyl)sulfanyl]-5-[4-(dimethylamino)phenyl]-1,3,4-oxadiazole (II) Geometric parameters (Å, º)

**Table:**

S1—C2	1.7317 (19)	C5A—H5AA	0.9300
S1—C7B	1.824 (2)	C6A—H6AA	0.9300
F1—C6B	1.357 (2)	C7A—H7AA	0.9600
Cl1—C2B	1.7346 (18)	C7A—H7AB	0.9600
N3—C2	1.278 (2)	C7A—H7AC	0.9600
N3—N4	1.418 (2)	C8A—H8AA	0.9600
N4—C5	1.286 (2)	C8A—H8AB	0.9600
N4A—C4A	1.369 (2)	C8A—H8AC	0.9600
N4A—C7A	1.440 (3)	C1B—C6B	1.381 (2)
N4A—C8A	1.442 (3)	C1B—C2B	1.398 (2)
O1—C2	1.361 (2)	C1B—C7B	1.500 (2)
O1—C5	1.372 (2)	C2B—C3B	1.378 (3)
C5—C1A	1.449 (2)	C3B—C4B	1.367 (3)
C1A—C6A	1.382 (3)	C3B—H3BA	0.9300
C1A—C2A	1.386 (3)	C4B—C5B	1.375 (3)
C2A—C3A	1.372 (3)	C4B—H4BA	0.9300
C2A—H2AA	0.9300	C5B—C6B	1.374 (3)
C3A—C4A	1.404 (3)	C5B—H5BA	0.9300
C3A—H3AA	0.9300	C7B—H7BA	0.9700
C4A—C5A	1.399 (3)	C7B—H7BB	0.9700
C5A—C6A	1.375 (3)		
			
C2—S1—C7B	100.11 (10)	N4A—C7A—H7AC	109.5
C2—N3—N4	105.48 (15)	H7AA—C7A—H7AC	109.5
C5—N4—N3	106.70 (15)	H7AB—C7A—H7AC	109.5
C4A—N4A—C7A	120.80 (19)	N4A—C8A—H8AA	109.5
C4A—N4A—C8A	120.70 (19)	N4A—C8A—H8AB	109.5
C7A—N4A—C8A	118.50 (19)	H8AA—C8A—H8AB	109.5
C2—O1—C5	102.31 (14)	N4A—C8A—H8AC	109.5
N3—C2—O1	113.54 (16)	H8AA—C8A—H8AC	109.5
N3—C2—S1	131.27 (14)	H8AB—C8A—H8AC	109.5
O1—C2—S1	115.19 (13)	C6B—C1B—C2B	114.82 (16)
N4—C5—O1	111.96 (16)	C6B—C1B—C7B	121.99 (17)
N4—C5—C1A	129.11 (17)	C2B—C1B—C7B	123.18 (16)
O1—C5—C1A	118.93 (15)	C3B—C2B—C1B	122.74 (17)
C6A—C1A—C2A	117.89 (17)	C3B—C2B—Cl1	118.33 (15)
C6A—C1A—C5	122.38 (16)	C1B—C2B—Cl1	118.91 (14)
C2A—C1A—C5	119.70 (17)	C4B—C3B—C2B	119.26 (19)
C3A—C2A—C1A	121.37 (18)	C4B—C3B—H3BA	120.4
C3A—C2A—H2AA	119.3	C2B—C3B—H3BA	120.4
C1A—C2A—H2AA	119.3	C3B—C4B—C5B	120.6 (2)
C2A—C3A—C4A	121.29 (18)	C3B—C4B—H4BA	119.7
C2A—C3A—H3AA	119.4	C5B—C4B—H4BA	119.7
C4A—C3A—H3AA	119.4	C6B—C5B—C4B	118.43 (19)
N4A—C4A—C5A	121.42 (18)	C6B—C5B—H5BA	120.8
N4A—C4A—C3A	121.92 (18)	C4B—C5B—H5BA	120.8
C5A—C4A—C3A	116.66 (17)	F1—C6B—C5B	117.80 (18)
C6A—C5A—C4A	121.41 (18)	F1—C6B—C1B	118.12 (18)
C6A—C5A—H5AA	119.3	C5B—C6B—C1B	124.07 (18)
C4A—C5A—H5AA	119.3	C1B—C7B—S1	114.87 (13)
C5A—C6A—C1A	121.35 (18)	C1B—C7B—H7BA	108.6
C5A—C6A—H6AA	119.3	S1—C7B—H7BA	108.6
C1A—C6A—H6AA	119.3	C1B—C7B—H7BB	108.6
N4A—C7A—H7AA	109.5	S1—C7B—H7BB	108.6
N4A—C7A—H7AB	109.5	H7BA—C7B—H7BB	107.5
H7AA—C7A—H7AB	109.5		
			
C2—N3—N4—C5	0.2 (2)	C2A—C3A—C4A—C5A	−1.4 (3)
N4—N3—C2—O1	−0.4 (2)	N4A—C4A—C5A—C6A	−178.3 (2)
N4—N3—C2—S1	179.84 (15)	C3A—C4A—C5A—C6A	2.1 (3)
C5—O1—C2—N3	0.4 (2)	C4A—C5A—C6A—C1A	−1.3 (3)
C5—O1—C2—S1	−179.82 (12)	C2A—C1A—C6A—C5A	−0.2 (3)
C7B—S1—C2—N3	−1.9 (2)	C5—C1A—C6A—C5A	178.09 (19)
C7B—S1—C2—O1	178.28 (13)	C6B—C1B—C2B—C3B	2.8 (2)
N3—N4—C5—O1	0.0 (2)	C7B—C1B—C2B—C3B	−178.83 (17)
N3—N4—C5—C1A	179.96 (18)	C6B—C1B—C2B—Cl1	−175.93 (12)
C2—O1—C5—N4	−0.2 (2)	C7B—C1B—C2B—Cl1	2.5 (2)
C2—O1—C5—C1A	179.82 (16)	C1B—C2B—C3B—C4B	−1.3 (3)
N4—C5—C1A—C6A	−176.9 (2)	Cl1—C2B—C3B—C4B	177.37 (15)
O1—C5—C1A—C6A	3.1 (3)	C2B—C3B—C4B—C5B	−0.7 (3)
N4—C5—C1A—C2A	1.4 (3)	C3B—C4B—C5B—C6B	1.0 (3)
O1—C5—C1A—C2A	−178.60 (17)	C4B—C5B—C6B—F1	−178.26 (17)
C6A—C1A—C2A—C3A	0.9 (3)	C4B—C5B—C6B—C1B	0.6 (3)
C5—C1A—C2A—C3A	−177.46 (19)	C2B—C1B—C6B—F1	176.46 (14)
C1A—C2A—C3A—C4A	−0.1 (3)	C7B—C1B—C6B—F1	−2.0 (2)
C7A—N4A—C4A—C5A	−178.7 (2)	C2B—C1B—C6B—C5B	−2.4 (2)
C8A—N4A—C4A—C5A	0.8 (3)	C7B—C1B—C6B—C5B	179.15 (17)
C7A—N4A—C4A—C3A	0.9 (3)	C6B—C1B—C7B—S1	−118.50 (17)
C8A—N4A—C4A—C3A	−179.6 (2)	C2B—C1B—C7B—S1	63.2 (2)
C2A—C3A—C4A—N4A	179.0 (2)	C2—S1—C7B—C1B	78.87 (15)

### 2-[(2-Chloro-6-fluorobenzyl)sulfanyl]-5-[4-(dimethylamino)phenyl]-1,3,4-oxadiazole (II) Hydrogen-bond geometry (Å, º)

*Cg*1 and *Cg*3 are the centroids of the O1/C2/N3/N4/C5 and C1*B*–C6*B* rings, respectively.

**Table:**

*D*—H···*A*	*D*—H	H···*A*	*D*···*A*	*D*—H···*A*
C2*B*—Cl1···*Cg*1^i^	1.74 (1)	3.30 (1)	4.939 (2)	156 (1)
C8*A*—H8*AB*···*Cg*3^ii^	0.96	2.94	3.857 (3)	161
C7*B*—H7*BA*···*Cg*3^iii^	0.97	2.85	3.674 (2)	143

Symmetry codes: (i) *x*, −*y*−1/2, *z*−3/2; (ii) −*x*+2, −*y*+1, −*z*+2; (iii) −*x*+1, −*y*+1, −*z*+1.
